# Protective effect of *Hemidesmus indicus* R.Br. root extract against cisplatin-induced cytogenetic damage in mouse bone marrow cells

**DOI:** 10.1590/S1415-47572010005000011

**Published:** 2010-03-01

**Authors:** Rajagopal Ananthi, Nallathambi Chandra, Sathiyavedu T. Santhiya

**Affiliations:** Department of Genetics, Dr. ALMPGIBMS, University of Madras, Tamil NaduIndia

**Keywords:** genoprotective, cytotoxic, chromosomal aberrations, micronucleus, mitotic index

## Abstract

The aqueous extract of *Hemidesmus indicus* roots was investigated for its *in vivo* antigenotoxic effect against cisplatin-induced cytogenetic damage. Swiss albino mice were administered with various doses of the extract either singly (50, 100 and 200 mg/kg body weight) or as split doses (10, 20 and 40 mg/kg bw/day) for five consecutive days by oral gavage. As endpoints, chromosome aberrations, micronuclei in polychromatic erythrocytes, mitotic index and PCE/NCE ratio were estimated. The extract protected the bone marrow cells from cisplatin-induced genotoxicity in an inverse dose-dependent manner. However, the extract was cytotoxic at all doses. But, under split dose regime it conferred a higher level of genoprotection and was not cytotoxic at the lower two doses. The presence of saponins, tannins, phenols, terpenoids, flavonoids and coumarins in the crude extract could explain these effects.

*Hemidesmus indicus* R.Br. (*Asclepiadaceae*) commonly known as Anantmul is a popular drug formulation used in the Ayurveda system of medicine. The roots served as remedy for leprosy, syphilis, leucoderma, asthma, dysentery, fever and, blood, kidney and urinary diseases (Kirtikar and Basu, 1984; Nadkarni, 1989) and root extracts have been found to exhibit various pharmacological properties (Aneja *et al.*, 2008; Austin, 2008; George *et al.*, 2008). A methanolic extract of *H.indicus* roots was recently shown to protect DNA from radiation-induced strand breaks and microsomal membranes from lipid peroxidation (Shetty *et al.*, 2005). Aqil *et al.* (2008) also demonstrated a genoprotective effect of a methanolic extract against methyl methane sulphonate- and sodium azide- induced mutagenicity in Salmonella tester strains TA 97a, TA 100, TA 102 and TA 104. The present investigation was aimed to evaluate the antigenotoxic activity of the aqueous extract of *H.indicus* roots against cisplatin-induced cytogenetic damage in mouse bone marrow cells.

The plant material was collected during summer from the Irula Tribal Women's Welfare Society, Chinglepet district, Tamil Nadu, India and authenticated. A voucher specimen (CASB H-6) was deposited at the Centre for Advanced Studies in Botany, University of Madras, Chennai, India. Shade-dried and powdered roots (300 g) were soaked in 3 liters of autoclaved distilled water for 48 h at 20 °C. The filtrate was condensed through a drying system to yield the extract (9.27%), which was stored at 4 °C until further use. Phytochemical screening of the extract to identify its active constituents was carried out using standard procedures (Harborne, 1973; Trease and Evans, 1989; Sofowara, 1993). Subsequently crude yield of the following constituents was determined: tannins (Van-Burden and Robinson, 1981), saponins (Obdoni and Ochuko, 2001), flavonoids (Bohm and Kocipai-Abyazan, 1974), alkaloids (Harborne, 1973) and phenols (Harborne, 1973; Obdoni and Ochuko, 2001). Coumarins and terpenoids were detected by a TLC method (British Pharmacopeia, 2007) and quenching zones were marked to be cut out and dissolved in 2 mL of methanol. Absorbance was read at 430 and 520 nm, respectively (Amenta, 1964).

Six- to eight- weeks-old (25 to 30 g) Swiss albino mice of both sexes were obtained from the Central Animal House Facility of the University of Madras, Taramani. Animals were maintained at 24 ± 2 °C in a controlled environment under a 12 h light/dark cycle with free access to standard laboratory feed pellets (Hindustan Lever Ltd., Bombay, India) and water *ad libitum.* The study was approved by the Animal Ethics Committee under CPCSEA, New Delhi, India. Evaluation of systemic toxicity was performed by the Up-and-Down method (OECD Guideline for Testing of Chemicals, 2001). The extract did not induce any mortality at 2,000 mg/kg body weight when given as a single dose. However, a significant reduction in mitotic index by 75% was observed at 550 mg/kg. Accordingly, lower doses of 50, 100, 200 mg/kg were chosen. The animals also received the extract at split doses of 10, 20 and 40 mg/kg/day for five consecutive days.

Swiss albino mice were segregated into experimental (N = 6) and control (N = 2) groups consisting of six mice each. Group 1 served as negative control and was given only distilled water. Cisplatin (Sigma – CAS No. 15663-27-1) at 5 mg/kg was administered intraperitoneally to animals representing positive control (group 2). Animals in groups 3, 4 and 5 received the aqueous extract by oral gavage at 50, 100 and 200 mg/kg respectively for evaluation of its mutagenic effect, if any. Cisplatin was given 2 h after treatment with the extract to animals in groups 6, 7, and 8 to determine its antimutagenic potential.

In the second experiment, mice were randomly distributed into seven groups consisting of six mice each. Mice were administered with the extract by gavage at the split doses of 10, 20 and 40 mg/kg/day for five consecutive days. In parallel, animals were injected intraperitoneally with cisplatin 2 h after treatment with the extract on the 5^th^ day. Animals administered with distilled water in parallel served as negative control. Both experimental and control animals were sacrificed 24 h later.

Bone marrow cells were processed to obtain slides for evaluation of chromosome aberrations by standard technique (OECD Guideline for Testing of Chemicals, 1997). One hundred metaphases per mouse were scored (at 100x magnification) to determine the percentage of chromosomal aberrations (CA). Gaps were excluded from analysis. The mitotic index was estimated by counting the metaphases over 5,000 cells per animal. The micronucleus assay was performed using the method based on OECD guidelines (OECD Guideline for Testing of Chemicals, 1997). The frequency of micronucleated polychromatic erythrocytes (MnPCEs) was determined by scoring 2,500 PCEs per animal. To evaluate the cytotoxic activity, simultaneous scoring of the number of normochromatic erythrocytes (NCEs) was done to determine PCE/NCE ratio.

Results were expressed as mean ± standard error (SE). Data were statistically analyzed using one-way ANOVA followed by Student-Neumann-Keul test. The level of significance was set at p < 0.05. The antigenotoxic potential of the extract was calculated by the formula proposed by Waters *et al.* (1990): %R = [(A - B)/(A - C)] x 100, where %R is the percent reduction, A is the CA or MN frequency upon treatment with cisplatin alone, B denotes the frequency upon treatment with the extract and cisplatin and C is the frequency with administration of distilled water.

The results obtained in CA and MN tests clearly demonstrated that *H.indicus* root extract was not genotoxic at the doses studied, as the frequencies of chromosomal aberrations and the polychromatic erythrocytes with micronuclei (MnPCEs) in mice treated with the extract did not differ significantly from those of the negative control (Table 1). However, the aqueous root extract significantly decreased the cisplatin-induced frequency of chromosomal aberrations in an inverse dose-dependent manner, with the maximum reduction being observed at the lowest dose of 50 mg/kg (p < 0.05). An inverse relationship between the frequency of MnPCEs and the administered dose of the extract further confirmed its genoprotective potential. Mitotic index and the PCE/NCE ratio, on the other hand, were significantly reduced by the extract at all doses (p < 0.05) (Table 1). The cytotoxic effect was greatly enhanced when cisplatin was administered subsequently.

Mice pretreated with split doses of the root extract for five consecutive days showed a statistically significant decrease in the frequencies of cisplatin-induced chromosomal aberrations and in MnPCEs, with the maximum reduction occurring at 20 mg/kg (p < 0.05) (Table 2). The extract itself, however, was not genotoxic at the three doses tested. It is of interest to note that the extract was also not cytotoxic at the lower doses of 10 and 20 mg/kg, although there was a significant reduction in mitotic index and PCE/NCE ratio at the higher dose of 40 mg/kg (p < 0.05) (Table 2). This ratio was found to further decrease upon subsequent treatment with cisplatin.

Root extracts of *H.indicus* have been shown to possess free radical scavenging and antioxidant properties (Ravishankara *et al.*, 2002; Mary *et al.*, 2003; Saravanan and Nalini, 2007). Since antioxidants are known as universal antimutagenic agents (Odin, 1997; Sarkar *et al.*, 1997; Giri *et al.*, 1998), the antigenotoxic effect of the root extract observed in this study could be attributed to these properties of its constituent bioactive compounds, such as 2-hydroxy-4-methoxy benzoic acid, lupeol acetate and terpenoids (Alam and Gomes, 1998; Chatterjee *et al.*, 2006; Kumar *et al.*, 2008). Quantitative estimation of crude chemical constituents in the aqueous extract of *Hemidesmus indicus* roots yielded the following results: tannins 3.06%, saponins 12.55%, flavonoids 1.12%, alkaloids 1.23%, terpenoids 0.79%, coumarins 0.91% and phenols 1.1%.

Kruawan and Kangsadalampai (2006) showed a correlation between the phenolic content of aqueous extracts from six herbs frequently consumed by Thai people and their antimutagenic potential. In another study, the antimutagenic effect of the fruits of *Terminalia chebula* (myrobalan) in *Allium cepa* root tip cells was suggested to be due to the presence of tannins, saponins and flavonoids (Rathore *et al.*, 2006). The cytotoxic activity of *H. indicus* root extract, which was also reported earlier by Thabrew *et al.* (2005), may be ascribed to the presence of coumarins (Kostova, 2005).

The present study showed that an aqueous extract of *Hemidesmus indicus* roots has potent antigenotoxic activity against cisplatin-induced cytogenetic damage and is cytotoxic. On the other hand, when administered in a split dose regime, it exhibited greater protection to the cells and was not cytotoxic at two lower doses. Studies to determine the safe dose of *H. indicus* extract are therefore desirable, as this root is very widely used in the treatment of several diseases and in herbal drinks in India. Besides characterization of active constituents and their evaluation, employing different test systems would aid in determining chemicals of potential therapeutic importance.

## Figures and Tables

**Table 1:** Effect of aqueous extract of *Hemidesmus indicus* roots on cisplatin-induced chromosomal aberrations, mitotic index, frequency of micronucleated erythrocytes and PCE/NCE ratio in mouse bone marrow cells.

Treatment	Dose (mg per kg)	Chromosomal aberrations (%)^*^	R (%)	Mitotic index	Frequency of MnPCEs per 2500 PCEs^*^	R (%)	PCE/NCE
Control	-	3.50 ± 0.22		4.05 ± 0.04	3.00 ± 0.26		1.02 ± 0.01
Cisplatin	5	57.17 ± 1.82^a^		1.48 ± 0.10^a^	32.00 ± 2.13^a^		0.71 ± 0.02^a^
Extract	50	3.33 ± 0.21		2.90 ± 0.06^a^	2.50 ± 0.22		0.92 ± 0.01^a^
	100	3.50 ± 0.22		2.46 ± 0.05^a^	2.67 ± 0.21		0.85 ± 0.01^a^
	200	3.67 ± 0.21		1.89 ± 0.03^a^	3.17 ± 0.31		0.79 ± 0.02^a^
Extract + Cisplatin	50	23.17 ± 1.01^b^	63.4	1.42 ± 0.04	10.50 ± 0.76^b^	74.1	0.68 ± 0.02
	100	27.17 ± 1.08^b^	55.9	1.26 ± 0.04^b^	13.50 ± 0.99^b^	63.8	0.52 ± 0.01^b^
	200	36.00 ± 1.07^b^	39.4	0.93 ± 0.03^b^	17.00 ± 1.06^b^	51.7	0.34 ± 0.02^b^

^*^Six animals per treatment. One-way ANOVA, p < 0.05 by Student Newman Keul test. ^a^ - Significant difference over control. ^b^ - Significant difference over cisplatin.

**Table 2:** Effect of split doses of aqueous extract of *Hemidesmus indicus* roots on cisplatin-induced chromosomal aberrations, mitotic index, frequency of micronucleated erythrocytes and PCE/NCE ratio in mouse bone marrow cells.

Treatment	Dose(mg per kg)	Chromosomal aberrations^*^	R (%)	Mitotic index	Frequency of MnPCEs per 2500 PCEs^*^	R (%)	PCE/NCE
Control	-	3.33 ± 0.33		4.32 ± 0.06	3.17 ± 0.31		1.03 ± 0.01
Cisplatin	5	57.17 ± 1.82^a^		1.48 ± 0.10^a^	32.0 ± 2.13^a^		0.71 ± 0.02^a^
Extract	10	3.17 ± 0.31		4.40 ± 0.16	2.33 ± 0.21		1.01 ± 0.03
	20	3.00 ± 0.26		4.21 ± 0.09	2.67 ± 0.21		0.99 ± 0.02
	40	3.50 ± 0.22		2.57 ± 0.14^a^	2.83 ± 0.17		0.87 ± 0.01^a^
Extract + Cisplatin	10	38.33 ± 1.54^b^	35.0	1.89 ± 0.03^b^	20.0 ± 0.97^b^	41.6	0.78 ± 0.01^b^
	20	13.83 ± 0.79^b^	80.5	2.32 ± 0.07^b^	8.00 ± 0.58^b^	83.3	0.83 ± 0.01^b^
	40	21.67 ± 0.88^b^	65.9	1.39 ± 0.08	11.5 ± 0.76^b^	71.1	0.67 ± 0.02

^*^Six animals per treatment. One-way ANOVA, p < 0.05 by Student Newman Keul test. ^a^ - Significant difference over control. ^b^ - Significant difference over cisplatin.
